# LncRNA FOXP4-AS1 Promotes the Progression of Esophageal Squamous Cell Carcinoma by Interacting With MLL2/H3K4me3 to Upregulate FOXP4

**DOI:** 10.3389/fonc.2021.773864

**Published:** 2021-12-14

**Authors:** Yunfeng Niu, Gaoyan Wang, Yan Li, Wei Guo, Yanli Guo, Zhiming Dong

**Affiliations:** ^1^ Laboratory of Pathology, Hebei Cancer Institute, The Fourth Hospital of Hebei Medical University, Shijiazhuang, China; ^2^ Experimental Center, Hebei University of Chinese Medicine, Shijiazhuang, China

**Keywords:** ESCC, FOXP4-AS1, long noncoding RNA, FOXP4, MLL2

## Abstract

Malignant tumors are a grave threat to human health. Esophageal squamous cell carcinoma (ESCC) is a common gastrointestinal malignant tumor. China has a high incidence of ESCC, and its morbidity and mortality are higher than the global average. Increasingly, studies have shown that long noncoding RNAs (lncRNAs) play a vital function in the occurrence and development of tumors. Although the biological function of FOXP4-AS1 has been demonstrated in various tumors, the potential molecular mechanism of FOXP4-AS1 in ESCC is still poorly understood. The expression of FOXP4 and FOXP4-AS1 was detected in ESCC by quantitative real-time PCR (qRT–PCR) or SP immunohistochemistry (IHC). shRNA was used to silence gene expression. Apoptosis, cell cycle, MTS, colony formation, invasion and migration assays were employed to explore the biological functions of FOXP4 and FOXP4-AS1. The potential molecular mechanism of FOXP4-AS1 in ESCC was determined by dual-luciferase reporter, RNA immunoprecipitation (RIP) and chromatin immunoprecipitation (ChIP). Here, we demonstrated that FOXP4-AS1 was significantly increased in ESCC tissues and cell lines, associated with lymph node metastasis and TNM staging. Cell function experiments showed that FOXP4-AS1 promoted the proliferation, invasion and migration ability of ESCC cells. The expression of FOXP4-AS1 and FOXP4 in ESCC tissues was positively correlated. Further research found that FOXP4-AS1, upregulated in ESCC, promotes FOXP4 expression by enriching MLL2 and H3K4me3 in the FOXP4 promoter through a “molecular scaffold”. Moreover, FOXP4, a transcription factor of β-catenin, promotes the transcription of β-catenin and ultimately leads to the malignant progression of ESCC. Finally, FOXP4-AS1 may be a new therapeutic target for ESCC.

## Introduction

Esophageal cancer ranks sixth in the global cause of death from malignant tumors (1). Esophageal squamous cell carcinoma (ESCC) accounts for the majority of esophageal cancers (2). China has a high incidence of ESCC, and its incidence has prominent geographical characteristics, especially in the Taihang Mountains of Linxian, Henan and Cixian, Hebei (3). The early symptoms of ESCC are atypical and insidious (4). Treatment is challenging since patients present with symptoms in the middle and late stages (5). Therefore, studying the molecular mechanisms of ESCC development and finding molecular markers for early diagnosis and prognostic assessment are essential means to improve the survival rate of patients.

LncRNAs, located in the nucleus or cytoplasm, are endogenous RNA molecules of more than 200 nucleotides (6). Nucleolar lncRNAs regulate chromatin modifications, transcription, mRNA stability, translation and posttranslational modifications (7). Cytoplasmic lncRNAs can competitively bind miRNAs and regulate miRNA target genes at the posttranscriptional level (8). A large amount of literature has shown that lncRNAs play a regulatory role in the development of ESCC (9–11). In this study, the differentially expressed lncRNA FOXP4-AS1 was screened by the microarrays. FOXP4-AS1, a member of the lncRNA family, is an antisense lncRNA of FOXP4. Extensive studies indicate that FOXP4-AS1 is highly expressed in several malignancies, including hepatocellular carcinoma (12), colorectal carcinoma (13), and nasopharyngeal carcinoma (14). Nevertheless, the role of FOXP4-AS1 in ESCC and its related molecular mechanisms have not been reported.

The purpose of this study was to detect the expression, correlation and regulatory mechanisms between FOXP4-AS1 and FOXP4. This study provides new theoretical evidence for targeted therapy of ESCC.

## Materials and Methods

### Bioinformatics Analysis

The GSE45670 and GSE161533 datasets were derived from the NCBI/GEO database to analyze differentially expressed lncRNAs in ESCC (https://www.ncbi.nlm.nih.gov/geo/). Prediction results are presented *via* Venny 2.1 (https://bioinfogp.cnb.csic.es/tools/venny/index.html). GEPIA (http://gepia.cancer-pku.cn/) was used to analyze FOXP4-AS1 expression in various cancers preliminarily. UCSC (http://genome.ucsc.edu/) was used to examine the abundance of H3K4me3 in the FOXP4 promoter. RPISeq (http://pridb.gdcb.iastate.edu/RPISeq/) was used to predict the binding ability of MLL2 with FOXP4-AS1. Animal TFDB3.0 (http://bioinfo.life.hust.edu.cn/AnimalTFDB/#!/) was used to find the motif sequence of FOXP4.

### Patients and Tissue Samples

With informed consent, tissue samples were collected from ESCC patients who underwent surgery at the Fourth Hospital of Hebei Medical University from 2015 to 2016. None of the patients received local or systemic treatment before surgery. Two pathologists confirmed the samples. A portion of the sample was placed in RNA later solution and stored at -80°C. Another portion was fixed in 10% neutral formalin and made into a wax block. The Ethics Committee of the Fourth Affiliated Hospital of Hebei Medical University accepted the study. The specific clinicopathological data of the patients are shown in **Table 1**.

**Table 1:** Clinicopathological data of esophageal squamous cell carcinoma.

Clinicopathological features	Case N. (%)
Gender	
Male	52 (75.4)
Female	17 (24.6)
Age	
≤61	29 (42.0)
>61	40 (58.0)
Histological grade	
Poor	15 (21.7)
Middle or high	54 (78.3)
Lymph node metastasis	
Negative (N0)	32 (46.4)
Positive (N1/N2/N3)	37 (53.6)
TNM stage	
I/II	37 (53.6)
III/IV	32 (46.4)

### Cell Culture

Human ESCC cell lines (Eca109, TE1, TE13, YES-2 and Kyse150) and esophageal normal epithelial cell lines (HEEpiCs) were acquired by the American Type Culture Collection. All cells were cultured in RPMI 1640 (Gibco) medium in 10% FBS (Invitrogen), and placed in an incubator (Thermo Fisher) at 37°C with 5% CO_2_.

### RNA Isolation and Quantitative Real-Time PCR (qRT–PCR)

Total RNA from tissues and cells was extracted using TRIzol reagent (Invitrogen) according to the protocol. cDNA was generated using the Transcriptor First Strand cDNA Synthesis Kit (Roche). qRT–PCR was performed on a StepOne Plus real-time quantitative PCR system using GoTaq^®^ qPCR Master Mix (Promega). The endogenous control of mRNA/lncRNA is ACTH. The 2^-ΔΔCT^ method was used to calculate RNA expression. Each sample was tested in triplicate, and the average was taken. The primer sequences used are in **Table 2**.

**Table 2:** Primers Used for Real-time PCR.

Names	Sequences(5’-3’)
FOXP4: Forward	GTGAGATGAGTCCCGCAGAG
FOXP4: Reverse	AGGCAGACTGTTTGCTGTCA
FOXP4-AS1: Forward	CCAGGTCTGCTGAAGATGTCA
FOXP4-AS1: Reverse	TACAGAGTGGCTTTCGAGCTG
MLL2: Forward	GGCGACATAGCCCGTAAGAC
MLL2: Reverse	CGTCCGCAGAGGTAGACAAG
E-cadherin: Forward	CGAGAGCTACACGTTCACGG
E-cadherin: Reverse	GGCCTTTTGACTGTAATCACACC
Vimentin: Forward	CGCCTGCAGGATGAGATTCAG
Vimentin: Reverse	TCAGGGAGGAAAAGTTTGGAAGA
β-catenin: Forward	GGCTACTGTTGGATTGATTC
β-catenin: Reverse	CCACAAATTGCTGCTGTGTC
ACTB: Forward	ACCGAGCGCGGCTACAG
ACTB: Reverse	CTTAATGTCACGCACGATTTCC
U6: Forward	CTCGCTTCGGCAGCACA
U6: Reverse	AACGCTTCACGAATTTGCGT
GAPDH: Forward	AGGTGAAGGTCGGAGTCAACG
GAPDH: Reverse	AGGGGTCATTGATGGCAACA

### Subcellular Fractionation

To clarify the cellular localization of FOXP4-AS1, cytoplasmic and nuclear fractions of Kyse150 and YES-2 cells (1×10^7^) were collected using the Nuclear/Cytolysis Fractionation Kit (BioVision) under the manufacturer’s guidelines. GAPDH and U6 were used as cytoplasmic/nuclear localization controls.

### Cell Transfection

The shRNAs (GenePharma) were designed to knockdown FOXP4-AS1, FOXP4 and MLL2. The sh-NC plasmid was included as a control. The overexpression plasmid was synthesized from GenScript, and the pcDNA3.1-NC plasmid was used as a control. Plasmids were transfected into cells in accordance with the Lipofectamine 2000 (Invitrogen). Transfected cells were used for qRT–PCR validation or further experiments.

### Cell Proliferation Assays

The MTS and colony formation assays were applied to detect the cell proliferation capacity. For the MTS assay, transfected cells were seeded into 96-well plates at 1×10^3^ per well. After 0, 24, 48, 72 and 96 hours of incubation 20 μl (500 μg/mL) MTS reagent was added to each well and incubated for 2 hours in the CO_2_ incubator. A Spark^®^ multimode microplate reader (Tecan) was used to measure the absorbance at 492 nm wavelength. For the colony formation assay, transfected cells were inoculated in six-well plates at 3×10^3^ cells per well and cultured for 7–14 days. Cells were fixed and stained with paraformaldehyde or 0.5% crystalline violet. A colony was a cluster of >50 cells, and the number of colonies was counted manually.

### Transwell Migration and Invasion Assays

The migration assays were executed using a nonmatrix gel-coated chamber (Corning) with an 8 μm pore membrane. A total of 1×10^5^ cells were inoculated into the upper chamber, and the lower chamber was 600 µl complete culture medium. After incubation for 24 hours, cells were fixed with paraformaldehyde and stained with crystal violet solution. Three areas were randomly selected for cell counting under a microscope (Leica). The invasion assay was performed as described above except for 50 µl Matrigel (Corning) in the upper chamber.

### Flow Cytometry Analysis

Apoptosis assays were executed using Annexin V-FITC/PI (BD Bioscience) double-staining reagent. Transfected cells were collected 48 hours later, washed twice with PBS and adjusted to 1×10^6^ cells per ml. Subsequently, cells were stained for apoptosis by adding 10 µl Annexin V-FITC and 5 µl PI under light-protected conditions. The cell cycle assays were performed using PI (Multi Science) single staining reagent. First, cells were treated with Triton X-100 and stained for 30 min with 500 µl PI at room temperature. Apoptosis and cell cycle were assessed by a flow cytometer (BD Bioscience).

### Western Blot

Cells were lysed in RIPA buffer (Solarbio) containing PMSF. Protein quantification was performed using the BCA Protein Quantification Kit (Solarbio). The protein sample was blended with the protein loading buffer and heated at 99°C for 5 minutes. The denatured proteins were separated by 10% SDS–PAGE and then transferred to a polyvinylidene difluoride (PVDF) membrane (Millipore). The PVDF membrane was placed in 5% skim milk for 1 hour and incubated at 4 ℃ overnight with the following primary antibodies: FOXP4 (1:1000, Abcam), GAPDH (1:10000, Abcam), MLL2 (1:1000, Abcam), E-cadherin (1:1000, Elabscience), vimentin (1:1000, Elabscience), and β-catenin (1:1000, Elabscience). Subsequently, the membranes were incubated with goat anti-rabbit IgG (KPL) for 1 hour, and then ECL reagent (Solarbio) was added for observation.

### Immunohistochemical Staining (IHC)

Paraffin-embedded tumor and normal tissues were sectioned and immunostained to determine FOXP4 protein expression using a rabbit anti-human FOXP4 antibody (1:1000, Abcam). Scoring criteria were evaluated according to previous reports (15). Three experienced pathologists, without knowledge of the clinical data, investigated the sections simultaneously.

### Vector Construction

Animal TFDB3.0 predicted the binding site of FOXP4 on the β-catenin promoter with two binding sites, and designed primers to amplify the fragment containing the binding site with upstream and downstream endonucleases HindIII and EcoRI. The fragments were ligated into pmirGL3-Basic and named pmirGL3-β-catenin-1 or pmirGL3-β-catenin-2. The mutant primers were devised in NEBase changer (http://nebasechanger.neb.com/) and synthesized by Sangon Biotech. The target fragments were successfully colony into pmir-GL3-Basic and named pmirGL3-β-catenin-1 (MUT) or pmirGL3-β-catenin-2 (MUT). The sequences of all constructed plasmids were sequenced in perspective.

### Dual-Luciferase Reporter Assay

PmirGL3-β-catenin-1/2(WT) and pmirGL3-β-catenin-1/2(MUT) were cotransfected with FOXP4 in Kyse150. A dual luciferase reporter system (Promega) was used to measure the luciferase activity of cells after 48 h.

### RNA Immunoprecipitation (RIP) Assay

The binding of FOXP4-AS1 to MLL2 was detected by RNA immunoprecipitation method using MLL2 antibody (Abcam) and the Magna RIP™ RNA-Binding Protein Immunoprecipitation Kit (Millipore) according to the manufacturer’s instructions. The IgG antibody was used as negative control. The purified RNA was subjected to qRT-PCR analysis.

### Chromatin Immunoprecipitation (ChIP)

ChIP assays were executed by the EZ-Magna ChIP kit (Millipore) and followed the manufacturer’s protocol. Cells were fixed in 4% PFA and quenched with glycine for 10 min. DNA was broken down to 200 to 600 bp using ultrasound. Immunoprecipitation of the lysate was performed with anti-H3K4me3 (Abcam), anti-MLL2 (Abcam), rabbit IgG or anti-FOXP4 (Abcam).

### Statistical Analysis

The experimental data for each one were performed individually and repeated three times. The results were statistically analyzed using SPSS 21.0 (IBM) and GraphPad Prism 8 (GraphPad), and shown as the mean ± SD. Student’s t-test and one-way ANOVA assessed significant differences between groups. Pearson correlation analysis was used to analyze the correlation between the FOXP4-AS1, FOXP4 and β-catenin. Survival curves were measured by the Kaplan–Meier method. Two-tailed values of *P*<0.05 were considered statistically significant..

## Results

### FOXP4-AS1 Was Upregulated in ESCC and Correlated With the Clinicopathological Features of ESCC

**Figure 1:**
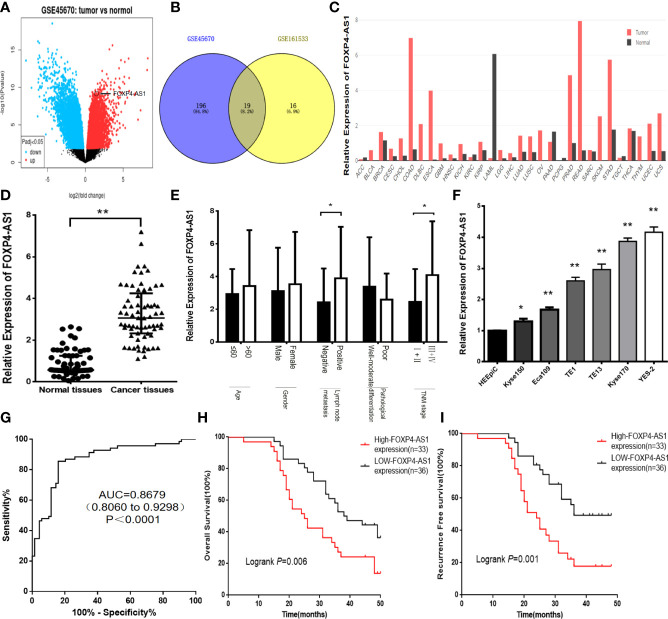
FOXP4-AS1 is upregulated in ESCC and correlates with clinicopathological data. **(A)** The differentially expressed lncRNAs in GSE45670 by the volcano plots (log2|FC|>1). **(B)** Highly expressed lncRNAs in GSE45670 and GSE161533. **(C)** FOXP4-AS1 expression in various tumors by GEPIA dataset. **(D–F)** The expression of FOXP4-AS1 in ESCC tissues, different subgroups and cell lines by qRT–PCR. **(G)** ROC analysis shows an AUC of 0.8679 for differentiating ESCC from normal tissue. OS **(H)** and RFS **(I)** of ESCC patients according to FOXP4-AS1 by Kaplan–Meier analysis. *p < .05 and **p < .01.

The differential lncRNAs in GSE45670 were screened by log|FC|>1 and *P*<0.05. A total of 457 differentially expressed lncRNAs were found, including 242 downregulated genes and 215 upregulated genes, among which the log|FC| value of FOXP4-AS1 was 2.08 (**Figure 1A**). We also found that FOXP4-AS1 was highly expressed in GSE161533 and GSE45670 (**Figure 1B**). The DEPIA revealed that FOXP4-AS1 was upregulated in most malignancies (**Figure 1C**). The qRT–PCR results indicated that FOXP4-AS1 was overexpressed in ESCC tissues (**Figure 1D**), and correlated with lymph node metastasis and TNM stage (**Figure 1E**). FOXP4-AS1 in six ESCC cell lines (Kyse150, YES-2, Kyse170, TE13, Eca109 and TE1) was considerably higher than the HEEpiC cells (**Figure 1F**). The expression of FOXP4-AS1 was highest in YES-2 cells, so YES-2 cells were used for knockdown experiments, and Kyse150 cells were used for overexpression experiments. Receiver operating characteristic (ROC) curve analysis showed that FOXP4-AS1 had a high-value area under the curve (AUC) of 0.8679 (0.8060 to 0.9298), a sensitivity of 85.50% and a specificity of 84.10% (**Figure 1G**) in distinguishing ESCC from normal tissues. Application of the Kaplan–Meier method revealed a significant reduction in overall survival (OS) (**Figure 1H**) and recurrence-free survival (RFS) (**Figure 1I**) in 69 ESCC patients with high expression of FOXP4-AS1. Collectively, these results suggest that FOXP4-AS1 may play a role as an oncogene in ESCC development, and may be a biomarker for ESCC diagnosis, treatment and prognosis.

### FOXP4-AS1 Promotes Malignant Biological Behavior in ESCC Cells

**Figure 2:**
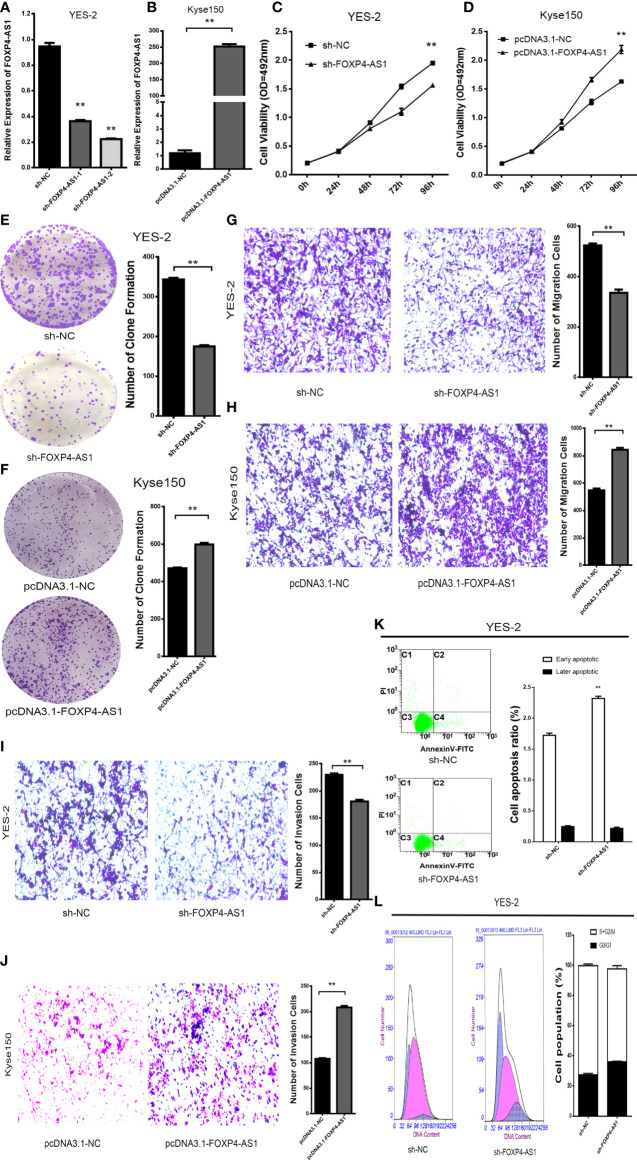
FOXP4-AS1 promotes the malignant progression of ESCC. Expression of FOXP4-AS1 in transfected sh-FOXP4-AS1 **(A)** or pcDNA3.1-FOXP4-AS1 **(B)** by qRT–PCR. **(C)** MTS assay showing the inhibition of YES-2 proliferation ability with sh-FOXP4-AS1. **(D)** Increased proliferative capacity of Kyse150 with pcDNA3.1-FOXP4-AS1 by MTS assay. **(E)** Colony formation assay indicates that the number of colony formation in YES-2 cells was reduced after FOXP4-AS1 deregulation. **(F)** Improved colony formation in Kyse150 cells transfected with pcDNA3.1-FOXP4-AS1. Transwell migration **(G)** and invasion assays **(I)** showing the decreased trans-cells of YES-2 with sh-FOXP4-AS1. Transwell migration **(H)** and invasion assays **(J)** demonstrated more Kyse150 trans-cells with pcDNA3.1-FOXP4-AS1. Apoptosis **(K)** and cycle **(L)** profiles of YES-2 cells transfected with sh-FOXP4-AS1 by FCM. **p < .01.

To evaluate the biological function of FOXP4-AS1 in ESCC. Knockdown of FOXP4-AS1 in YES-2 using shRNA, we chose sh-FOXP4-AS1–2 for follow-up experiments because it exhibited relatively strong knockdown efficiency (**Figure 2A**). Transfection of pcDNA3.1-FOXP4-AS1 into Kyse150 greatly increased FOXP4-AS1 compared to the empty vector control (pcDNA3.1-NC) (**Figure 2B**). MTS and colony formation assays revealed that the proliferation ability of YES-2 cells transfected with sh-FOXP4-AS1 was markedly inhibited (**Figures 2C, E**). The proliferation ability of Kyse150 cells transfected with pcDNA3.1-FOXP4-AS1 was enhanced (**Figures 2D, F**). Consistent with the migration and invasion assays, fewer trans-well cells were discovered in the sh-FOXP4-AS1 group than the sh-NC group (**Figures 2G, I**). At the same time, transfection of pcDNA3.1-FOXP4-AS1 in Kyse150 had the opposite effect (**Figures 2H, J**). Flow cytometry was used to detect the effect of FOXP4-AS1 on the ESCC cell cycle and apoptosis. The data showed that the early apoptotic cells in the sh-FOXP4-AS1 group grew in number (**Figure 2K**). The cell number of G0/G1 phase was augmented, and S+G2/M phase was reduced in YES-2 cells transfected with sh-FOXP4-AS1 (**Figure 2L**).

### FOXP4 Was Increased in ESCC and Positively Correlated With FOXP4-AS1

**Figure 3:**
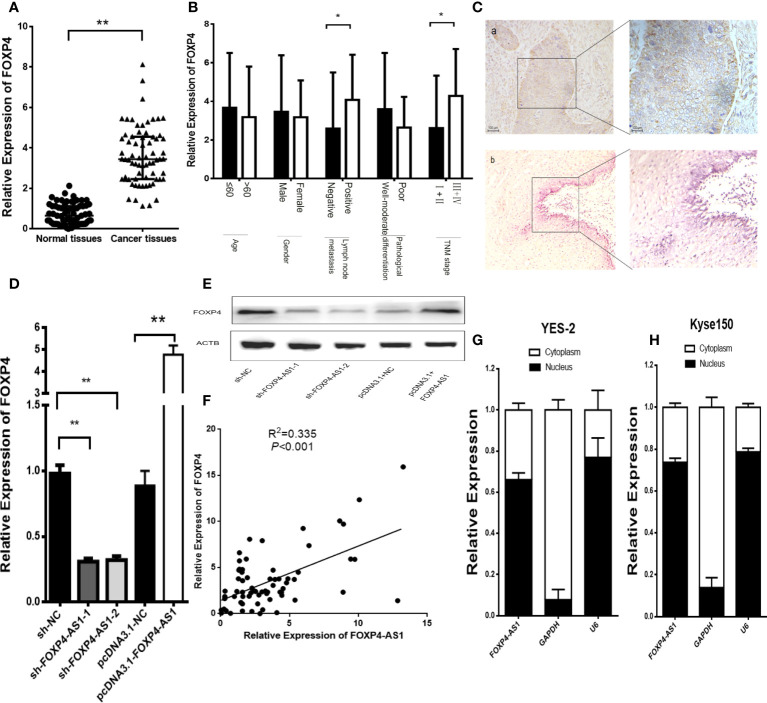
FOXP4 is highly expressed in ESCC and increased by FOXP4-AS1 at the mRNA and protein levels. The expression of FOXP4 in ESCC tissues **(A)** and different subgroups **(B)** by qRT–PCR. **(C)** FOXP4 protein in ESCC by IHC (SP Left×200; Right×400). a: ESCC tissue; b normal tissue. FOXP4 mRNA **(D)** and protein **(E)** levels with FOXP4-AS1 by qRT–PCR or WB. **(F)** The correlation between FOXP4-AS1 and FOXP4 by qRT–PCR. Subcellular localization of FOXP4-AS1 in YES-2 **(G)** and Kyse150 **(H)**, GAPDH and U6 were used as cytoplasmic and nuclear controls. *p < .05 and **p < .01.

FOXP4 mRNA and protein were dramatically highly expressed in ESCC, and correlated with lymph node metastasis and TNM stage (**Figures 3A–C**). Knockdown of FOXP4-AS1 in YES-2 drastically reduced FOXP4 mRNA and protein. Overexpression of FOXP4-AS1 at Kyse150 promoted an increase in FOXP4 (**Figures 3D, E**; **S1A**). Pearson correlation analysis revealed that FOXP4-AS1 positively correlated with FOXP4 mRNA in ESCC (**Figure 3F**). To further investigate the possible molecular mechanisms of FOXP4-AS1 involvement in cancer development, we examined its subcellular localization. FOXP4-AS1 was mainly located in the nucleus of ESCC cells (**Figures 3G, H**).

### FOXP4-AS1 Regulates the Expression of FOXP4 Through MLL2

**Figure 4:**
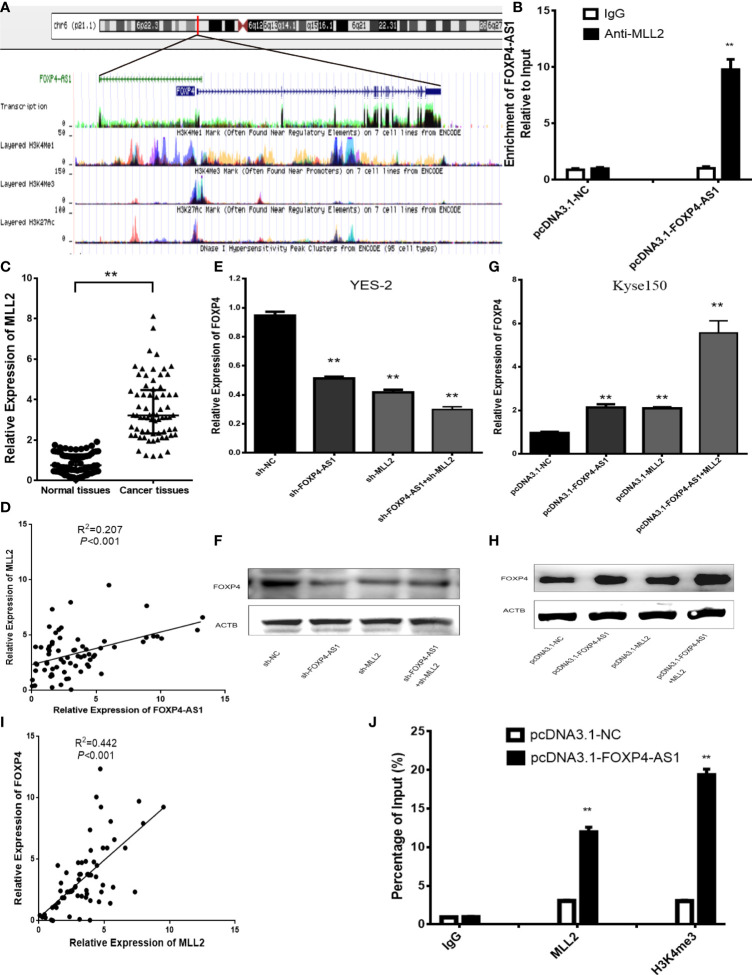
FOXP4-AS1 upregulates FOXP4 expression through interacting with MLL2. **(A)** Epistatic regulatory maps of FOXP4 and FOXP4-AS1 by UCSC. **(B)** The binding of FOXP4-AS1 to MLL2 in Kyse150 by RIP. **(C)** The expression of MLL2 in ESCC by qRT–PCR. **(D)** The association between FOXP4-AS1 and MLL2 by qRT–PCR. FOXP4 mRNA **(E, G)** and protein **(F, H)** by coexpressing FOXP4-AS1 and MLL2. **(I)** The correlation between FOXP4-AS1 and MLL2 by the qRT–PCR. **(J)** FOXP4-AS1 increased MLL2 and H3K4me3 enrichment in the FOXP4 promoter as determined by ChIP assay. **p < .01.

Numerous studies have shown that antisense lncRNAs can cis-regulate sense mRNAs’ expression and molecular mechanism (16–18). Overexpression of FOXP4-AS1 in Kyse150 cells upregulated the mRNA and protein of FOXP4. We considered whether FOXP4-AS1, located in the nucleus, might regulate FOXP4 by binding to some nuclear factors, such as transcription factors or epigenetic regulatory enzymes. We observed that the promoter of FOXP4 was enriched in H3K4me3 signaling, implying that FOXP4 might be affected by histone methylation (**Figure 4A**). Myeloid/lymphoid or mixed-lineage leukemia (MLL) family genes are essential enzymes for modifying H3K4 methylation, so we speculate that FOXP4-AS1 may regulate FOXP4 by binding to MLL family genes. The binding capacity of MLL1-MLL5 to FOXP4-AS1 was predicted by RPISeq, showing that MLL2 binds optimally to FOXP4-AS1 (RF:0.70, SVM:0.95). RIP assays confirmed that FOXP4-AS1 could bind to the MLL2 protein (**Figure 4B**). MLL2 was highly expressed in ESCC (**Figure 4C**), and positively correlated with FOXP4-AS1 in ESCC by Pearson analysis (**Figure 4D**). Moreover, MLL2 overexpression increased the mRNA and protein of FOXP4. MLL2 and FOXP4-AS1 cotransfection increased FOXP4 at the transcriptional and translational levels, indicating that MLL2 and FOXP4-AS1 have synergistic effects on FOXP4 regulation (**Figures 4E–H** **;** **S1B, C**). In addition, FOXP4 was positively associated with MLL2 in ESCC (**Figure 4I**). ChIP assay data further demonstrated that upregulated FOXP4-AS1 promoted the enrichment of MLL2 and H3K4me3 in the FOXP4 promoter (**Figure 4J**). However, MLL2 did not affect the expression of FOXP4-AS1 (**Figures S2A, B**). Thus, FOXP4-AS1 enriched MLL2 and H3K4me3 in the FOXP4 promoter, which induced transcription of FOXP4.

### FOXP4 Affects the Malignant Biological Behavior of ESCC Cells

**Figure 5:**
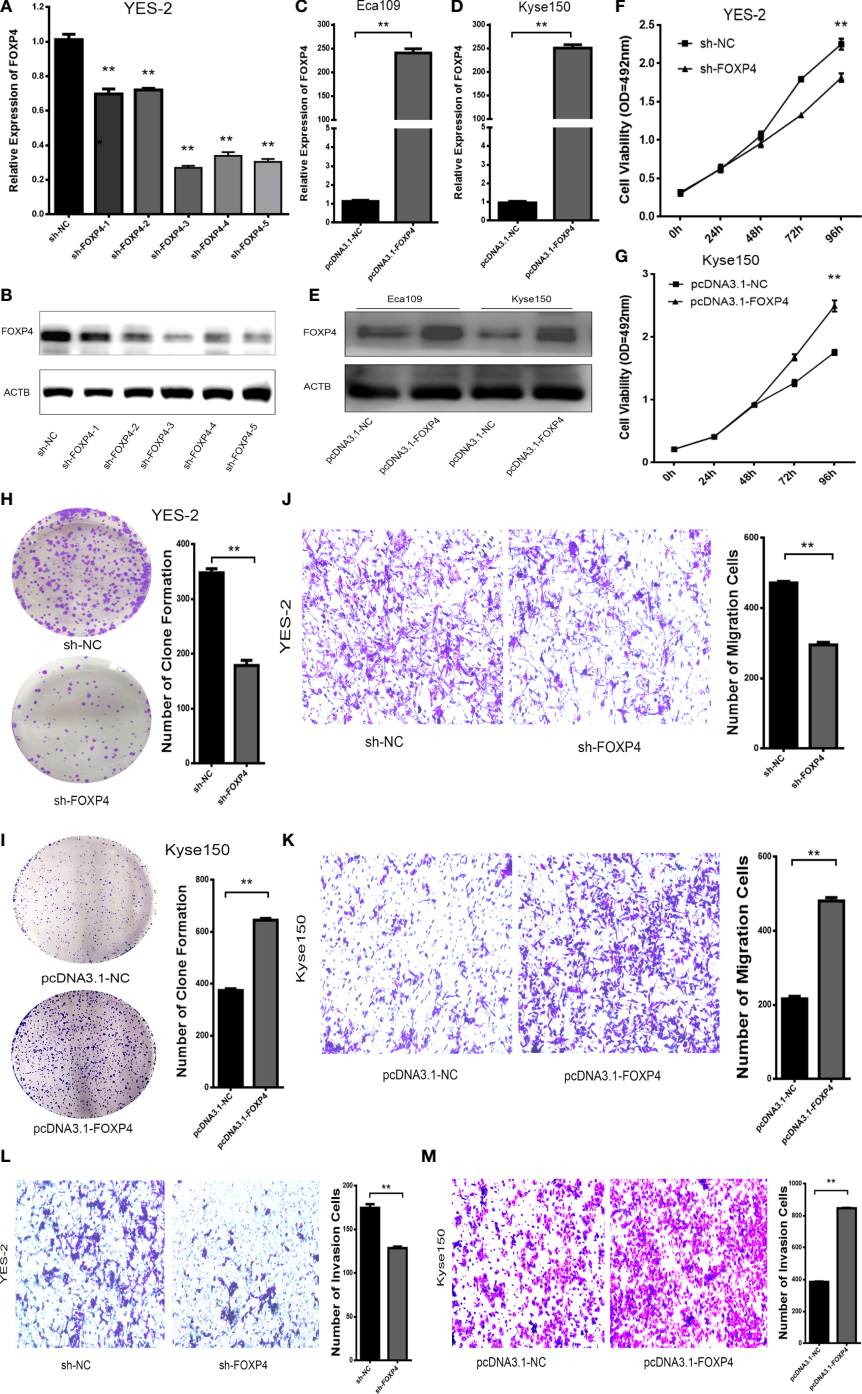
FOXP4 promotes the malignant progression of ESCC. The expression of FOXP4 mRNA **(A, C, D)** and protein **(B, E)** in transfected sh-FOXP4 or pcDNA3.1-FOXP4 by qRT–PCR or WB. **(F)** MTS assay showing the inhibition of YES-2 proliferation ability with sh-FOXP4. **(G)** Increased proliferative capacity of Kyse150 with pcDNA3.1-FOXP4 by MTS assay. **(H)** Colony formation assay indicated that deregulated FOXP4 follows the lower colony formation in YES-2 cells. **(I)** Enhanced colony formation in Kyse150 cells with pcDNA3.1-FOXP4. Transwell migration **(J)** and invasion assays **(K)** show the decreased trans-cells of YES-2 with sh-FOXP4. Transwell migration **(L)** and invasion assay **(M)** demonstrated more Kyse150 trans-cells with pcDNA3.1-FOXP4. **p < .01.

The effect of FOXP4 on the biological function of ESCC was examined by knocking down FOXP4 with shRNAs in YES-2 cells. We chose sh-FOXP4–3 for subsequent experiments, due to its relatively more robust knockdown efficiency (**Figures 5A, B** **;** **S1D**). Transfection of pcDNA3.1-FOXP4 in Kyse150 resulted in substantially higher mRNA and protein levels of FOXP4 than pcDNA3.1-NC (**Figures 5C–E** **;** **S1E**). The proliferation ability of YES-2 cells transfected with sh-FOXP4 was greatly inhibited as shown by MTS and colony formation assays (**Figures 5F, H**). Transfection with pcDNA3.1-FOXP4 substantially enhanced the proliferation ability of Kyse150 cells (**Figures 5G, I**). Migration and invasion assays had consistent results, with fewer trans-well cells in the sh-FOXP4 group than the sh-NC group (**Figures 5J, L**). While transfection of pcDNA3.1-FOXP4 had the opposite effect (**Figures 5K, M**).

### FOXP4 Can Act as an Upstream Transcription Factor of β-Catenin

**Figure 6:**
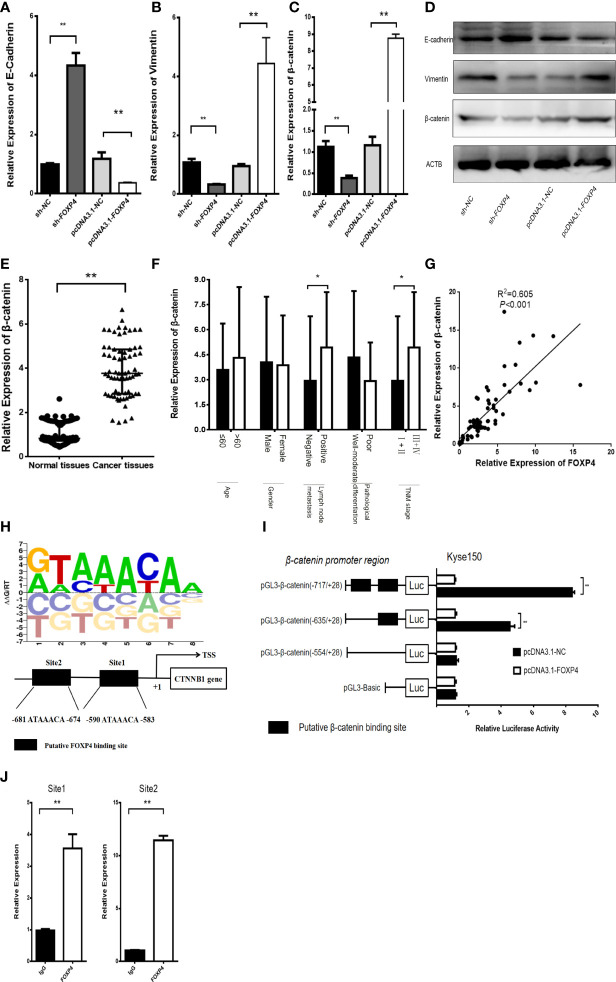
FOXP4 regulates β-catenin expression as a transcription factor. The mRNA **(A, B, C)** and protein **(D)** levels of EMT‐related genes (E‐cadherin, β-catenin, and Vimentin) with sh‐FOXP4 or pcDNA3.1-FOXP4 by qRT–PCR or WB. The expression of β-catenin in ESCC **(E)** and different subgroups **(F)** by qRT–PCR. **(G)** The correlation between FOXP4 and β-catenin by qRT–PCR. **(H)** The predicted FOXP4 binding site sequence in the promoter of β-catenin. **(I)** The effect of FOXP4 on the luciferase activity of β-catenin by dual-luciferase reporter assay. **(J)** The direct interaction of FOXP4 with the β-catenin promoter by ChIP. *p < .05 and **p < .01.

EMT plays a critical role in the invasion and distant metastasis of malignant tumors, including ESCC (19–21). We aimed to clarify the function of FOXP4 in mediating EMT in ESCC. qRT–PCR and WB showed that the expression of vimentin and β-catenin was considerably lower in the sh-FOXP4 group than the control group, and overexpression of FOXP4 increased their expression, while E-cadherin had the opposite effect (**Figures 6A–D** **;** **S1F–H**). We found that β-catenin was upregulated in ESCC (**Figure 6E**), and correlated with lymph node metastasis and TNM stage (**Figure 6F**). Pearson analysis revealed that FOXP4 is related to β-catenin in ESCC (**Figure 6G**). Animal TFDB3.0 predicted that FOXP4 could act as a transcription factor of β-catenin, and had two binding sites (**Figure 6H**). The luciferase reporter assay demonstrated that FOXP4 increased the luciferase activity of pmirGL3-β-catenin-1/2 (WT) in Kyse150 cells, whereas the mutant did not (**Figure 6I**). ChIP results showed that the expression of PCR-amplified products was substantially higher in the FOXP4 antibody group than the control group, suggesting that FOXP4 had binding site activity with the β-catenin promoter (**Figure 6J**). However, the promoter region of FOXP4-AS1 did not have a binding site for FOXP4, and the experimental results indicated that FOXP4 did not affect FOXP4-AS1 (**Figures S2C, D**). Therefore, we inferred that FOXP4 promoted β-catenin transcription by binding to the β-catenin promoter.

### FOXP4-AS1 Regulates the β-Catenin Expression and ESCC Progression *via* the MLL2/FOXP4 Axis

**Figure 7:**
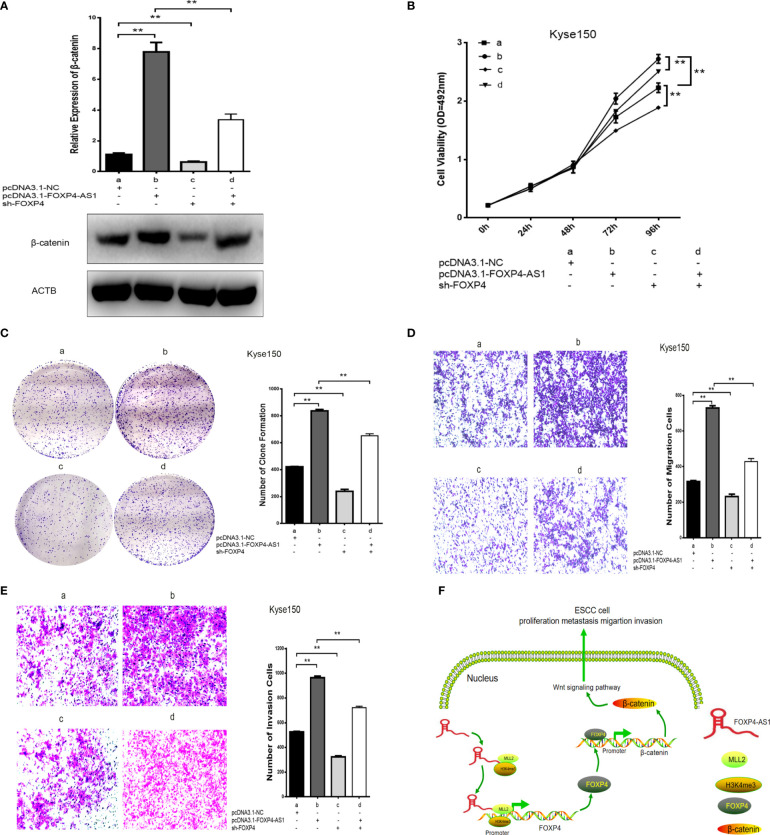
Coexpression of FOXP4-AS1 and FOXP4 promotes β-catenin expression and ESCC progression. **(A)** The mRNA and protein levels of β-catenin with FOXP4-AS1 and FOXP4 by qRT‐PCR or WB. The effect of FOXP4-AS1 and FOXP4 coexpression on ESCC cell proliferation by MTS **(B)** or Colony formation **(C)** assay. FOXP4-AS1 and FOXP4 coexpression on ESCC cell migration **(D)** and invasion **(E)** by Transwell assay. **(F)** The possible molecular mechanisms for the involvement of FOXP4-AS1 in ESCC progression. **p < .01.

We found that FOXP4 acts as a transcription factor for β-catenin to verify further whether FOXP4-AS1 regulates β-catenin expression and ESCC biological function through the MLL2/FOXP4 axis. FOXP4-AS1/FOXP4 knockdown and overexpression plasmids were constructed and cotransfected into Kyse150 cells. It was revealed that FOXP4-AS1 promoted β-catenin expression, and the highest expression of β-catenin was observed cotransfection with FOXP4-AS1/FOXP4. However, sh-FOXP4-AS1/FOXP4 eliminated this effect (**Figure 7A** **;** **S1I**). Cell function experiments showed that cotransfection of FOXP4-AS1/FOXP4 increased the proliferation, migration and invasion ability of ESCC cells. Conversely, sh-FOXP4-AS1/FOXP4 partially rescued this effect (**Figures 7B–E**). These results suggest that FOXP4-AS1 regulates β-catenin expression and biological functions through the MLL2/FOXP4 axis (**Figure 7F**).

## Discussion

Esophageal squamous cell carcinoma (ESCC) is a common malignancy worldwide, ranking sixth in cancer-related deaths (22). With the development of medical technology, there has been a tremendous improvement in the treatment of ESCC, but its 5-year survival rate is still low (23). It remains insufficient to guide the treatment and prognosis of ESCC (24). In recent years, cancer alterations at the molecular level have attracted researchers’ attention (25). LncRNAs are transcripts longer than 200 bp which cannot encode proteins, and play a crucial role in malignant tumor development, such as tumor cell proliferation, differentiation, programmed death, invasion and lymph node metastasis (26). FOXP4-AS1 is a member of the lncRNA family and antisense lncRNAs of FOXP4 (27). FOXP4-AS1 was an oncogene in most cancers (28–30). Li et al. (31). found that FOXP4-AS1 was upregulated in colorectal cancer, and correlated with TNM stage and tumor size. Knockdown of FOXP4-AS1 restrained cell proliferation, tumor growth and induced apoptosis. Yang et al. (32). proposed that FOXP4-AS1 was overexpressed in osteosarcoma (OS) and an independent prognostic risk factor for OS. FOXP4-AS1 promoted OS cell proliferation, migration and cell cycle, but inhibited apoptosis. Furthermore, FOXP4-AS1 downregulated LATS1 by binding to LSD1 and EZH2, which promoted OS development and progression. Zhong et al. (33). corroborated the poor prognosis of highly expressed FOXP4-AS1 in nasopharyngeal carcinoma (NPC). FOXP4-AS1 upregulated STMN1 through its interaction with miR-423–5p, acting as a ceRNA to promote NPC progression. Liu et al. (34). found that high expression of FOXP4-AS1 in pancreatic ductal adenocarcinoma (PDAC) tissue was related to poorer medical outcomes. They determined the specific molecular mechanism of FOXP4-AS1 in PDAC and its two targeted therapeutics by multiple genome-wide approaches.

This paper analyzed the differential lncRNAs of GSE161533 and GSE45670, and found that FOXP4-AS1 was upregulated in ESCC. We demonstrated that FOXP4-AS1 was substantially increased in ESCC, and correlated considerably with prognostic and clinicopathological features, including lymph node metastasis and TNM staging. FOXP4-AS1 was a biomarker for ESCC diagnosis due to its high AUC value, and act as an oncogene to promote the progression of ESCC. In addition, FOXP4 was a sense transcript of FOXP4-AS1. We confirmed that FOXP4 was upregulated in ESCC and positively correlated with FOXP4-AS1. FOXP4-AS1 augmented FOXP4 mRNA and protein expression.

The forkhead family regulates embryonic development, cell cycle and tumorigenesis. Each member has a highly conserved structural domain of approximately 100 amino acids (35). Numerous studies have shown that forkhead proteins can act as transcription factors to regulate the transcription of target genes (36–38). FOXP4, a member of the P subfamily of the forkhead box (FOX) family, is a novel transcription factor (39). FOXP4 is located on human chromosome 6p21 and can regulate tumor growth, progression and metastasis (40). We verified that FOXP4 could promote the malignant progression of ESCC as an oncogene.

Antisense lncRNAs account for approximately 50–70% of lncRNAs (41). In recent years, the role of antisense lncRNAs in malignancies, including ESCC, has been increasingly studied (42–44). However, how antisense lncRNAs regulate the sense mRNAs deserves in-depth exploration (45). Dang et al. (46). concluded that AFAP1-AS1, a novel biomarker for gastric cancer (GC), promoted the malignant biological behavior of GC cells and acted as a ceRNA to target AFAP1 by sponging miR-205–5p. Wang et al. (47). found that STXBP5-AS1, an antioncogene, suppressed the expression of STXBP5 in non-small cell lung cancer (NSCLC) by blocking PI3K/AKT, thereby inhibiting the progression of NSCLC. Wang et al. (48). determined that FAM83A-AS1 upregulated FAM83A by enhancing the stability of FAM83A pre-mRNA, and promoted tumorigenesis of lung adenocarcinoma (LUAD). Zhou et al. (49). demonstrated that NCK1-AS1 promoted lung squamous cell carcinoma progression (LUSC) by upregulating NCK1 through interaction with MYC. It has been shown that antisense lncRNAs play a cis-regulatory role in sense mRNAs (50–52). Antisense lncRNAs located in the nucleus can recruit DNA, histone-modifying enzymes, or transcription factors to specific sites to cis-regulate the expression of sense mRNAs (53). Our study demonstrated that MLL2, a member of the MLL family and a component of the ASC-2/NCOA6 complex (ASCOM), has histone methylation activity and thus participates in transcriptional activation (54). The FOXP4-AS1 located in the nucleus could increase the expression of FOXP4 by interacting with MLL2. FOXP4-AS1 increased the enrichment of MLL2 and H3K4me3 at the FOXP4 promoter, which induced FOXP4 transcription.

Epithelial-mesenchymal transition (EMT), the property of epithelial cells to acquire mesenchymal cells, is necessary to develop invasion and metastasis of malignant tumors (55, 56). During the development of EMT in malignant tumors, epithelial cell markers (e.g., E-cadherin) are decreased, in contrast, mesenchymal cell markers (e.g., Vimentin) and EMT-associated transcription factors (e.g., β-catenin) are increased (57). Our results showed that FOXP4 upregulated vimentin and β-catenin, as well as downregulated E-cadherin, suggesting that FOXP4 promoted EMT in ESCC. Meanwhile, β-catenin, encoded by CTNNB1, is a crucial factor in the WNT signaling pathway and regulates tumorigenesis development (58). It has been shown that β-catenin was upregulated in ESCC and positively correlated with FOXP4. FOXP4 acted as a transcription factor of β-catenin to promote the transcription of β-catenin. Interestingly, FOXP4-AS1/FOXP4 promoted the malignant progression of ESCC by regulating β-catenin, which provided new research directions for the diagnosis, treatment, and prognosis of ESCC.

## Conclusions

In summary, we showed that FOXP4-AS1, upregulated in ESCC, promotes FOXP4 expression by enriching MLL2 and H3K4me3 in the FOXP4 promoter through a “molecular scaffold”. Moreover, FOXP4, a transcription factor of β-catenin, promotes the transcription of β-catenin and ultimately leads to the malignant progression of ESCC. Thus, FOXP4-AS1 provides a new research target for the diagnosis, treatment, and prognosis of ESCC.

## Data Availability Statement

The original contributions presented in the study are included in the article/**Supplementary Material**. Further inquiries can be directed to the corresponding author.

## Ethics Statement

The Ethics Committee approved this study of the Fourth Hospital of Hebei Medical University, and informed consent was obtained from each participant.

## Author Contributions

YN conceived the experimental design and wrote the manuscript. GW and YL performed the experiments. WG and YG counted the data. ZD made the graphs. All authors read and approved the final manuscript.

## Funding

This study was supported by the Natural Science Foundation of Hebei Province, China (No. H2019206664).

## Conflict of Interest

The authors declare that the research was conducted in the absence of any commercial or financial relationships that could be construed as a potential conflict of interest.

## Publisher’s Note

All claims expressed in this article are solely those of the authors and do not necessarily represent those of their affiliated organizations, or those of the publisher, the editors and the reviewers. Any product that may be evaluated in this article, or claim that may be made by its manufacturer, is not guaranteed or endorsed by the publisher.
