# Corrigendum: LncRNA FOXP4-AS1 promotes the progression of esophageal squamous cell carcinoma by interacting with MLL2/H3K4me3 to upregulate FOXP4

**DOI:** 10.3389/fonc.2022.1041732

**Published:** 2022-10-14

**Authors:** Yunfeng Niu, Gaoyan Wang, Yan Li, Wei Guo, Yanli Guo, Zhiming Dong

**Affiliations:** ^1^ Laboratory of Pathology, Hebei Cancer Institute, The Fourth Hospital of Hebei Medical University, Shijiazhuang, China; ^2^ Experimental Center, Hebei University of Chinese Medicine, Shijiazhuang, China

**Keywords:** ESCC, FOXP4-AS1, long noncoding RNA, FOXP4, MLL2

In the original article, there was a mistake in **Figure 1** as published. In **Figure 1B**, “GSE151633” should be “GSE161533”. The corrected **Figure 1** appears below.

**Figure 1:**
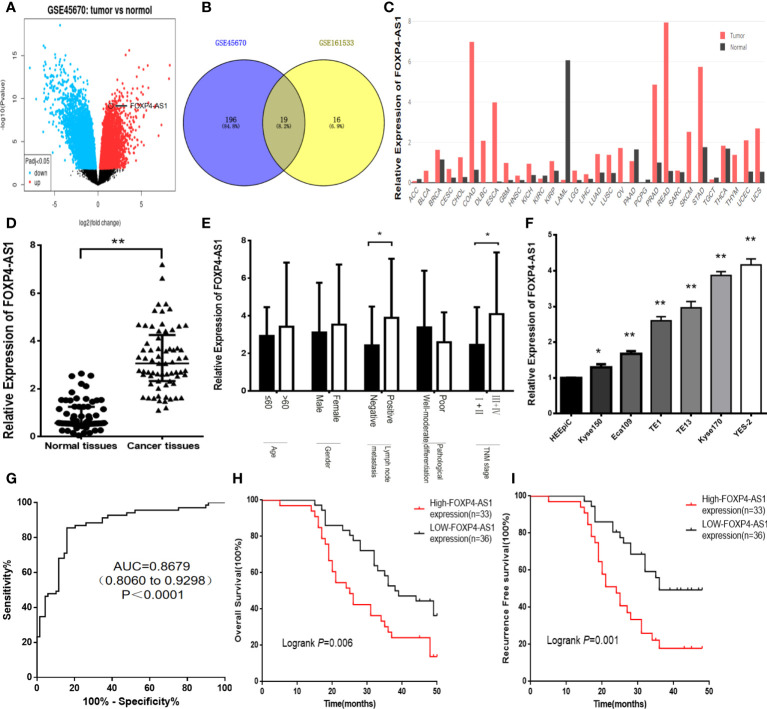
FOXP4-AS1 is upregulated in ESCC and correlates with clinicopathological data. **(A)** The differentially expressed lncRNAs in GSE45670 by the volcano plots (log2|FC|>1). **(B)** Highly expressed lncRNAs in GSE45670 and GSE161533. **(C)** FOXP4-AS1 expression in various tumors by GEPIA dataset. **(D–F)** The expression of FOXP4-AS1 in ESCC tissues, different subgroups and cell lines by qRT–PCR. **(G)** ROC analysis shows an AUC of 0.8679 for differentiating ESCC from normal tissue. OS **(H)** and RFS **(I)** of ESCC patients according to FOXP4-AS1 by Kaplan–Meier analysis. *p < .05 and **p < .01.

In addition, there was a mistake in **Figure 2** as published. In **Figure 2F**, the original image of the clone formation experiment has been reworked. The corrected **Figure 2** appears below.

**Figure 2:**
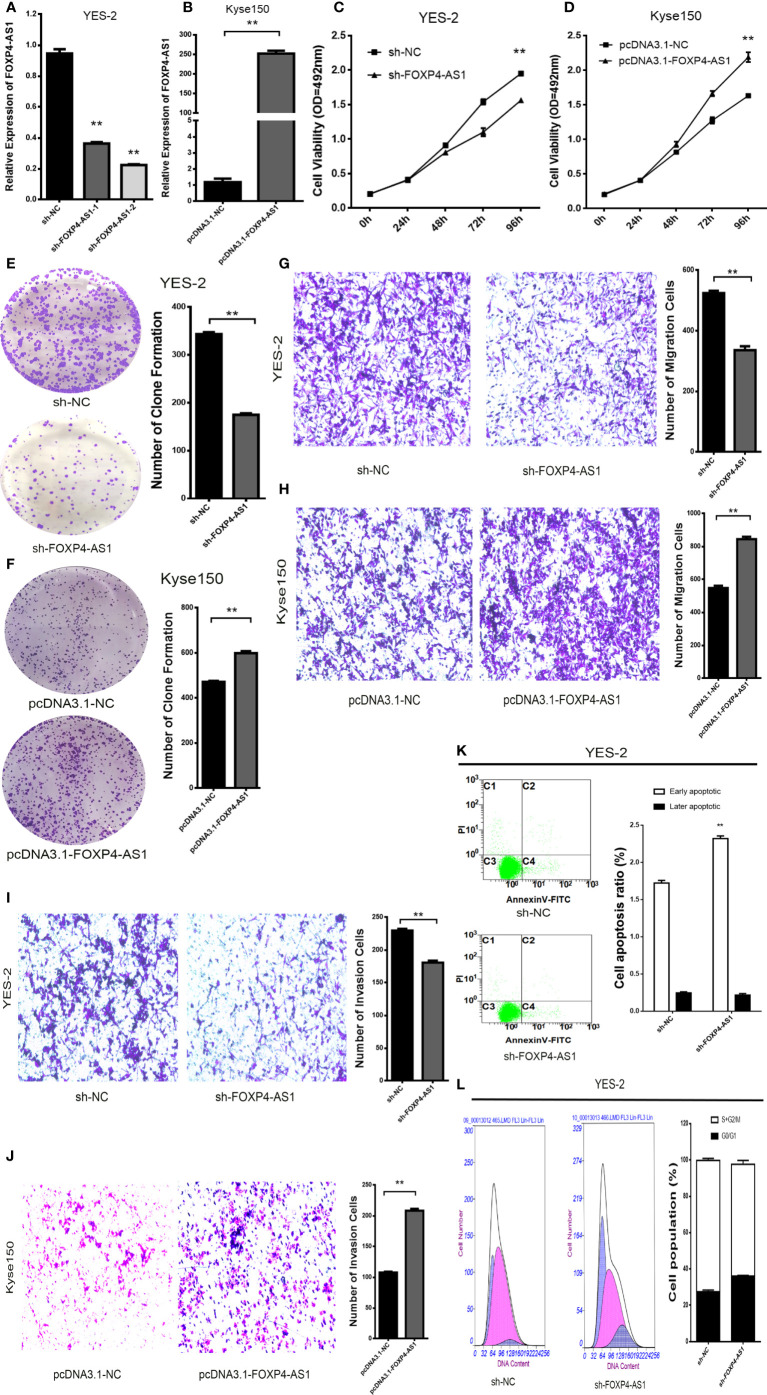
FOXP4-AS1 promotes the malignant progression of ESCC. Expression of FOXP4-AS1 in transfected sh-FOXP4-AS1 **(A)** or pcDNA3.1-FOXP4-AS1 **(B)** by qRT–PCR. **(C)** MTS assay showing the inhibition of YES-2 proliferation ability with sh-FOXP4-AS1. **(D)** Increased proliferative capacity of Kyse150 with pcDNA3.1-FOXP4-AS1 by MTS assay. **(E)** Colony formation assay indicates that the number of colony formation in YES-2 cells was reduced after FOXP4-AS1 deregulation. **(F)** Improved colony formation in Kyse150 cells transfected with pcDNA3.1-FOXP4-AS1. Transwell migration **(G)** and invasion assays **(I)** showing the decreased trans-cells of YES-2 with sh-FOXP4-AS1. Transwell migration **(H)** and invasion assays **(J)** demonstrated more Kyse150 trans-cells with pcDNA3.1-FOXP4-AS1. Apoptosis **(K)** and cycle **(L)** profiles of YES-2 cells transfected with sh-FOXP4-AS1 by FCM. **p < .01.

In addition, there was a mistake in **Figure 3** as published. In **Figure 3F**, “Relative expression of FOXP4-AS1” has been changed to “Relative Expression of FOXP4-AS1”. #In Figure 3A, the title of the Y axis has changed from "Expression of FOXP4 (2-△△CT)" to "Relative Expression of FOXP4". The corrected **Figure 3** appears below.

**Figure 3:**
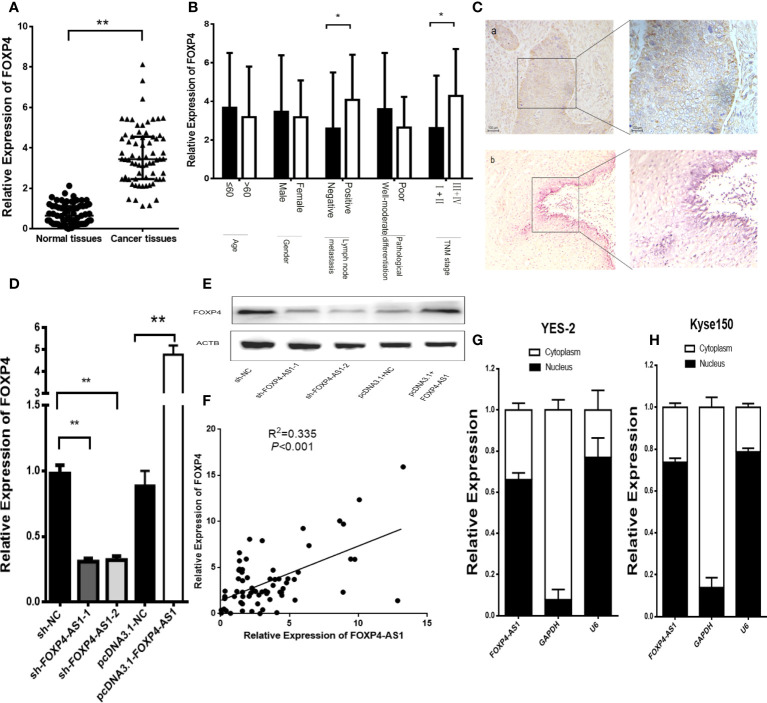
FOXP4 is highly expressed in ESCC and increased by FOXP4-AS1 at the mRNA and protein levels. The expression of FOXP4 in ESCC tissues **(A)** and different subgroups **(B)** by qRT–PCR. **(C)** FOXP4 protein in ESCC by IHC (SP Left×200; Right×400). a: ESCC tissue; b normal tissue. FOXP4 mRNA **(D)** and protein **(E)** levels with FOXP4-AS1 by qRT–PCR or WB. **(F)** The correlation between FOXP4-AS1 and FOXP4 by qRT–PCR. Subcellular localization of FOXP4-AS1 in YES-2 **(G)** and Kyse150 **(H)**, GAPDH and U6 were used as cytoplasmic and nuclear controls. *p < .05 and **p < .01.

In addition, there was a mistake in **Figure 4** as published. In **Figure 4D**, “Relative expression of FOXP4-AS1” has been changed to “Relative Expression of FOXP4-AS1”. In Figure 4C, the title of the Y axis has changed from “Expression of MLL2 (2-△△CT)" to “Relative Expression of MLL2”. The corrected **Figure 4** appears below.

**Figure 4:**
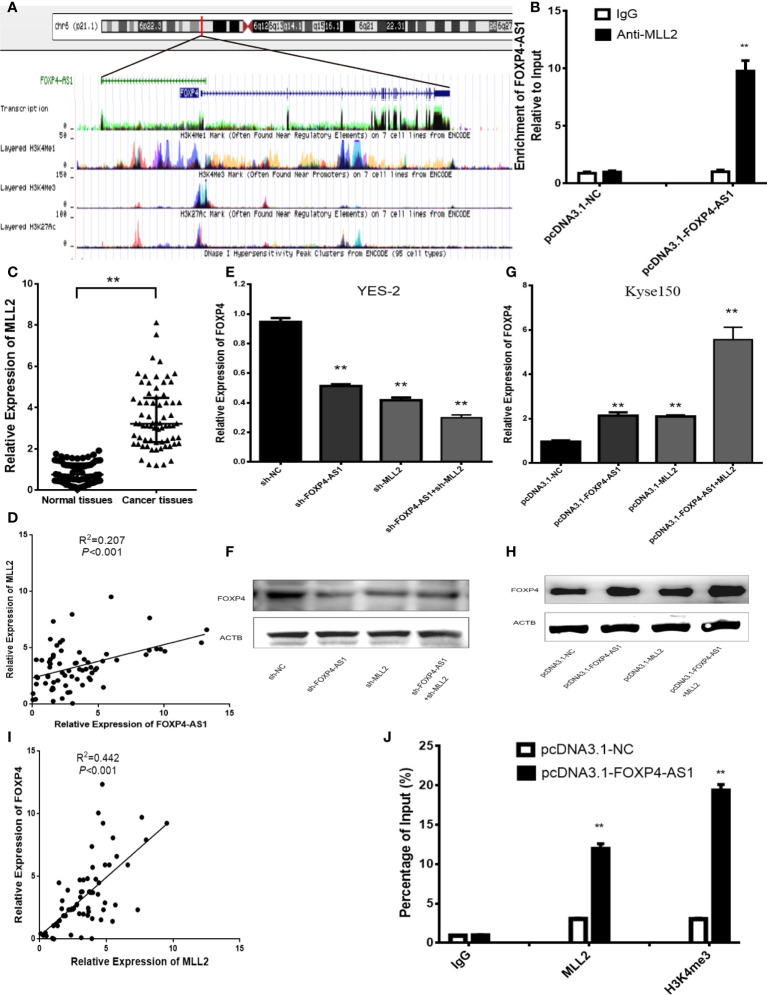
FOXP4-AS1 upregulates FOXP4 expression through interacting with MLL2. **(A)** Epistatic regulatory maps of FOXP4 and FOXP4-AS1 by UCSC. **(B)** The binding of FOXP4-AS1 to MLL2 in Kyse150 by RIP. **(C)** The expression of MLL2 in ESCC by qRT–PCR. **(D)** The association between FOXP4-AS1 and MLL2 by qRT–PCR. FOXP4 mRNA **(E, G)** and protein **(F, H)** by coexpressing FOXP4-AS1 and MLL2. **(I)** The correlation between FOXP4-AS1 and MLL2 by the qRT–PCR. **(J)** FOXP4-AS1 increased MLL2 and H3K4me3 enrichment in the FOXP4 promoter as determined by ChIP assay. **p < .01.

In addition, there was a mistake in **Figure 5** as published. In **Figure 5I**, the original image of the clone formation experiment has been reworked. The corrected **Figure 5** appears below.

**Figure 5:**
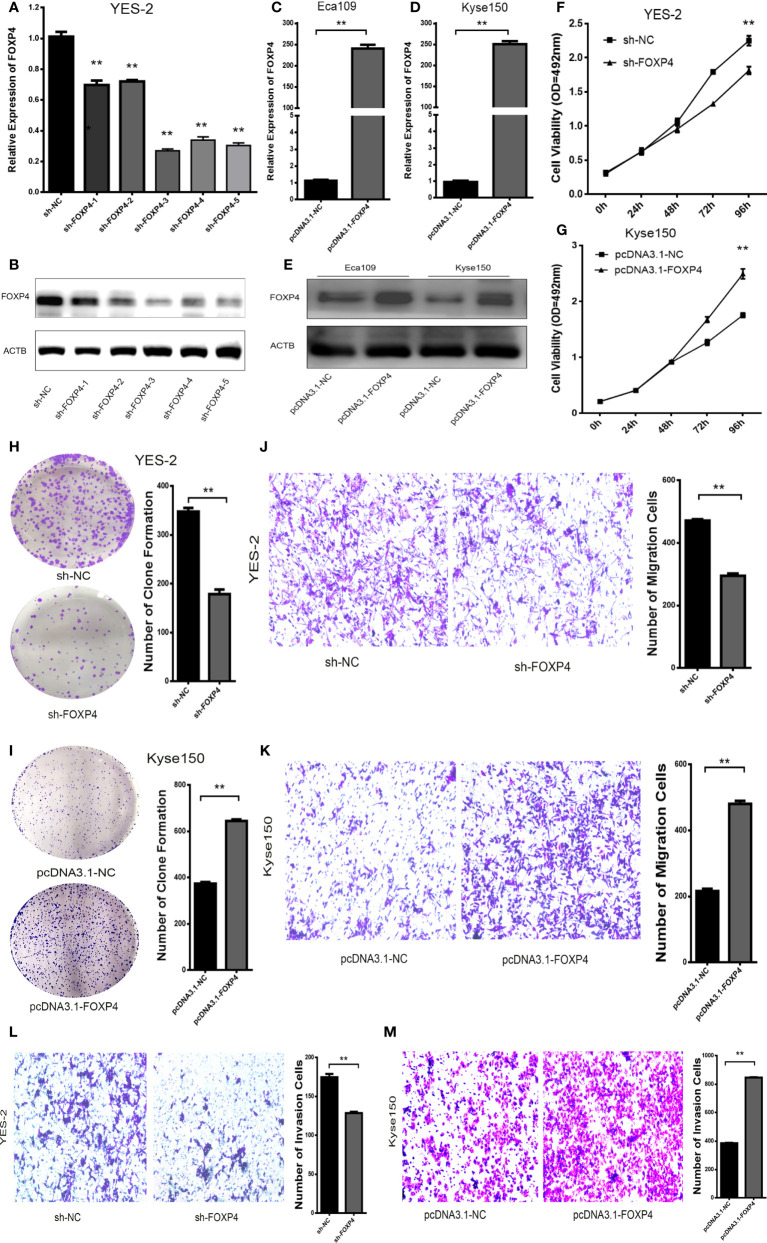
FOXP4 promotes the malignant progression of ESCC. The expression of FOXP4 mRNA **(A, C, D)** and protein **(B, E)** in transfected sh-FOXP4 or pcDNA3.1-FOXP4 by qRT–PCR or WB. **(F)** MTS assay showing the inhibition of YES-2 proliferation ability with sh-FOXP4. **(G)** Increased proliferative capacity of Kyse150 with pcDNA3.1-FOXP4 by MTS assay. **(H)** Colony formation assay indicated that deregulated FOXP4 follows the lower colony formation in YES-2 cells. **(I)** Enhanced colony formation in Kyse150 cells with pcDNA3.1-FOXP4. Transwell migration **(J)** and invasion assays **(K)** show the decreased trans-cells of YES-2 with sh-FOXP4. Transwell migration **(L)** and invasion assay **(M)** demonstrated more Kyse150 trans-cells with pcDNA3.1-FOXP4. **p < .01.

In addition, there was a mistake in **Figure 6** as published. In **Figure 6I**, the luciferase reporter gene experiment was reworked. The corrected **Figure 6** appears below.

**Figure 6:**
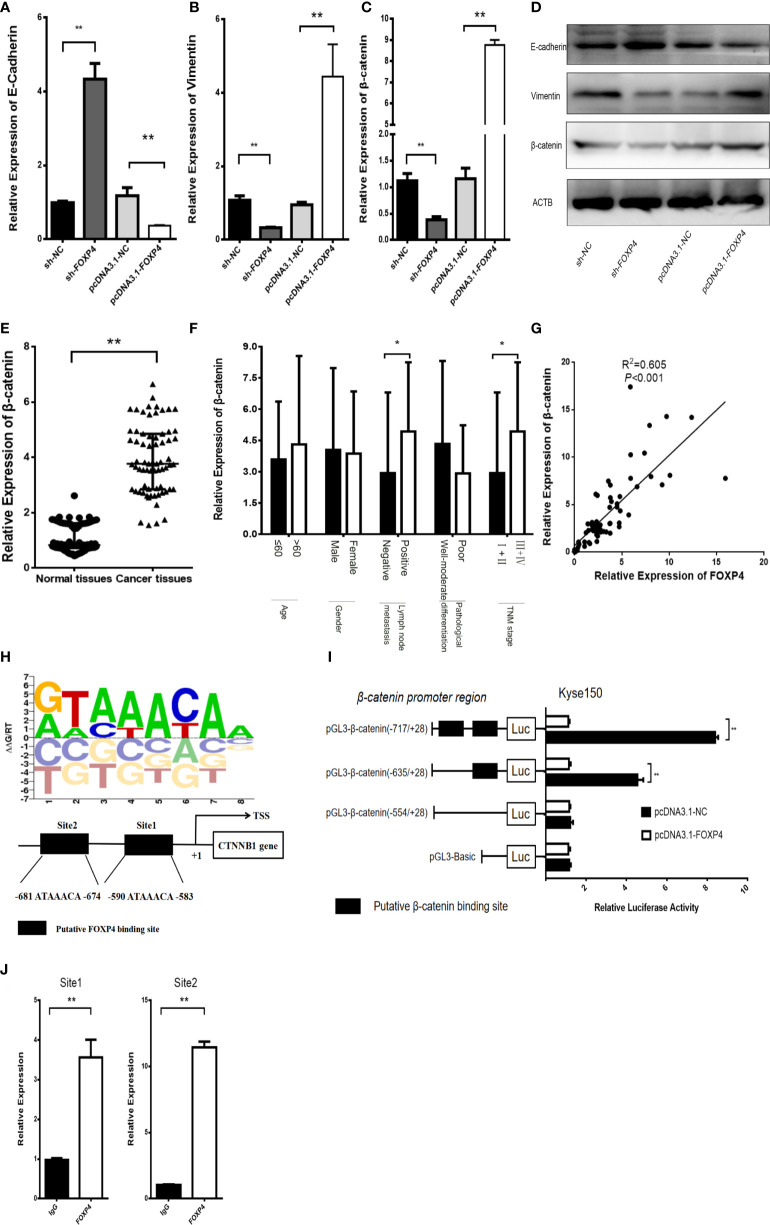
FOXP4 regulates β-catenin expression as a transcription factor. The mRNA **(A–C)** and protein **(D)** levels of EMT‐related genes (E‐cadherin, β-catenin, and Vimentin) with sh‐FOXP4 or pcDNA3.1-FOXP4 by qRT–PCR or WB. The expression of β-catenin in ESCC **(E)** and different subgroups **(F)** by qRT–PCR. **(G)** The correlation between FOXP4 and β-catenin by qRT–PCR. **(H)** The predicted FOXP4 binding site sequence in the promoter of β-catenin. **(I)** The effect of FOXP4 on the luciferase activity of β-catenin by dual-luciferase reporter assay. **(J)** The direct interaction of FOXP4 with the β-catenin promoter by ChIP. *p < .05 and **p ≶ .01.

In addition, there was a mistake in **Figure 7** as published. In **Figure 7**, the overall experiment was divided into four groups. The overexpression of FOXP4-AS1 promoted metastasis and invasion of esophageal squamous carcinoma cells, however transfection of pcDNA3.1-FOXP4-AS1 and sh-FOXP4 attenuated the ability of transfection of pcDNA3.1-FOXP4-AS1 alone to metastasize and invade esophageal squamous carcinoma cells. The corrected **Figure 7** appears below.

**Figure 7:**
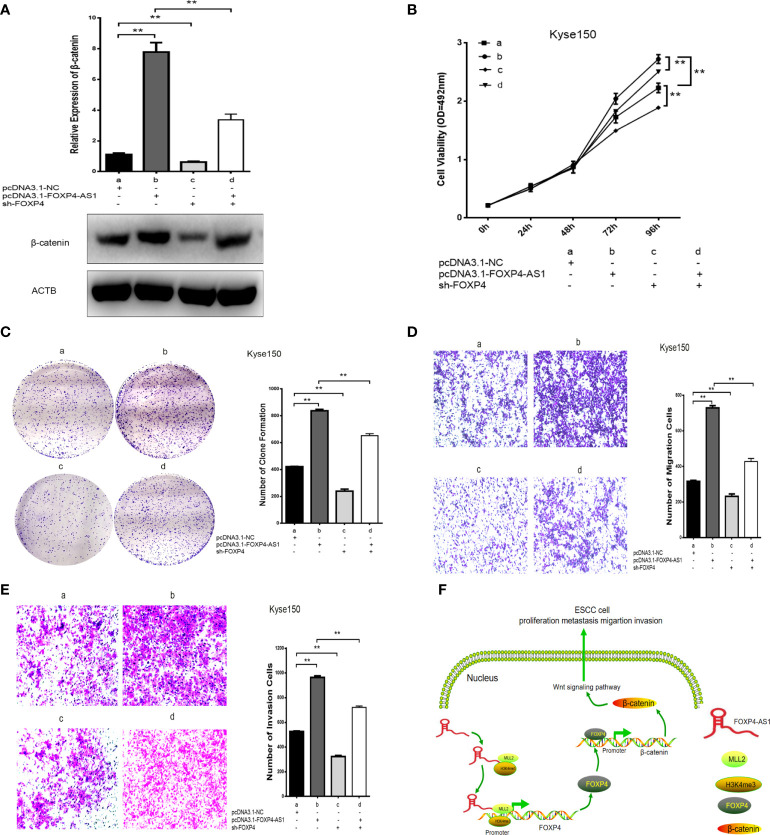
Coexpression of FOXP4-AS1 and FOXP4 promotes β-catenin expression and ESCC progression. **(A)** The mRNA and protein levels of β-catenin with FOXP4-AS1 and FOXP4 by qRT‐PCR or WB. The effect of FOXP4-AS1 and FOXP4 coexpression on ESCC cell proliferation by MTS **(B)** or Colony formation **(C)** assay. FOXP4-AS1 and FOXP4 coexpression on ESCC cell migration **(D)** and invasion **(E)** by Transwell assay. **(F)** The possible molecular mechanisms for the involvement of FOXP4-AS1 in ESCC progression. **p < .01.

In addition, there was a mistake in Figure S1 as published. In Figure S1I, “groups” should be “We divided the group into four groups”. The updated file may be viewed via the original article.

The authors apologize for these errors and state that they do not change the scientific conclusions of the article in any way. The original article has been updated.

## Publisher’s note

All claims expressed in this article are solely those of the authors and do not necessarily represent those of their affiliated organizations, or those of the publisher, the editors and the reviewers. Any product that may be evaluated in this article, or claim that may be made by its manufacturer, is not guaranteed or endorsed by the publisher.

