# Supplementary material for: LncRNA FOXP4-AS1 Promotes the Progression of Esophageal Squamous Cell Carcinoma by Interacting With MLL2/H3K4me3 to Upregulate FOXP4
Source: Front Oncol. 2021 Dec 14;11:773864. doi: 10.3389/fonc.2021.773864 (PMC8712759; doi:10.3389/fonc.2021.773864)
Supplement: Supplementary file 1 [file DataSheet_1.docx]

**SUPPLEMENTARY FIGURES**


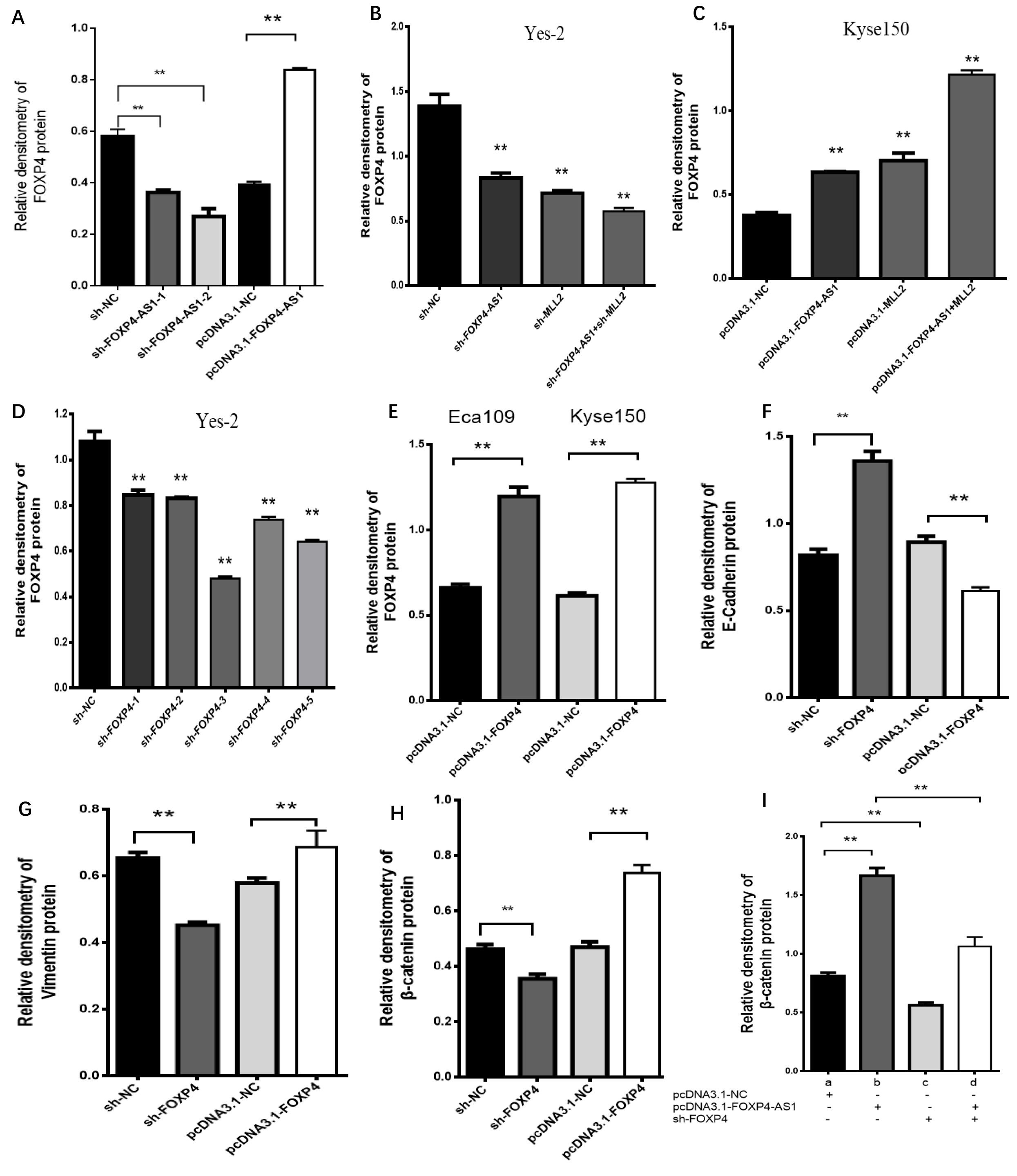


**FIGURE S1 |** Relative densitometry of proteins. **(A)** Relative densitometry of FOXP4 protein with FOXP4-AS1 by WB. **(B,C)** Relative densitometry of FOXP4 protein by coexpressing FOXP4-AS1 and MLL2. **(D,E)** Relative densitometry of FOXP4 protein in transfected sh-FOXP4 or pcDNA3.1-FOXP4 by WB. **(F,G,H)** The relative protein densitometry of EMT-related genes (E-cadherin, β-catenin, and Vimentin) with sh-FOXP4 or pcDNA3.1-FOXP4 by WB. **(I)** Relative densitometry of β-catenin protein with FOXP4-AS1 and FOXP4 by WB. ***p* < .01.

**
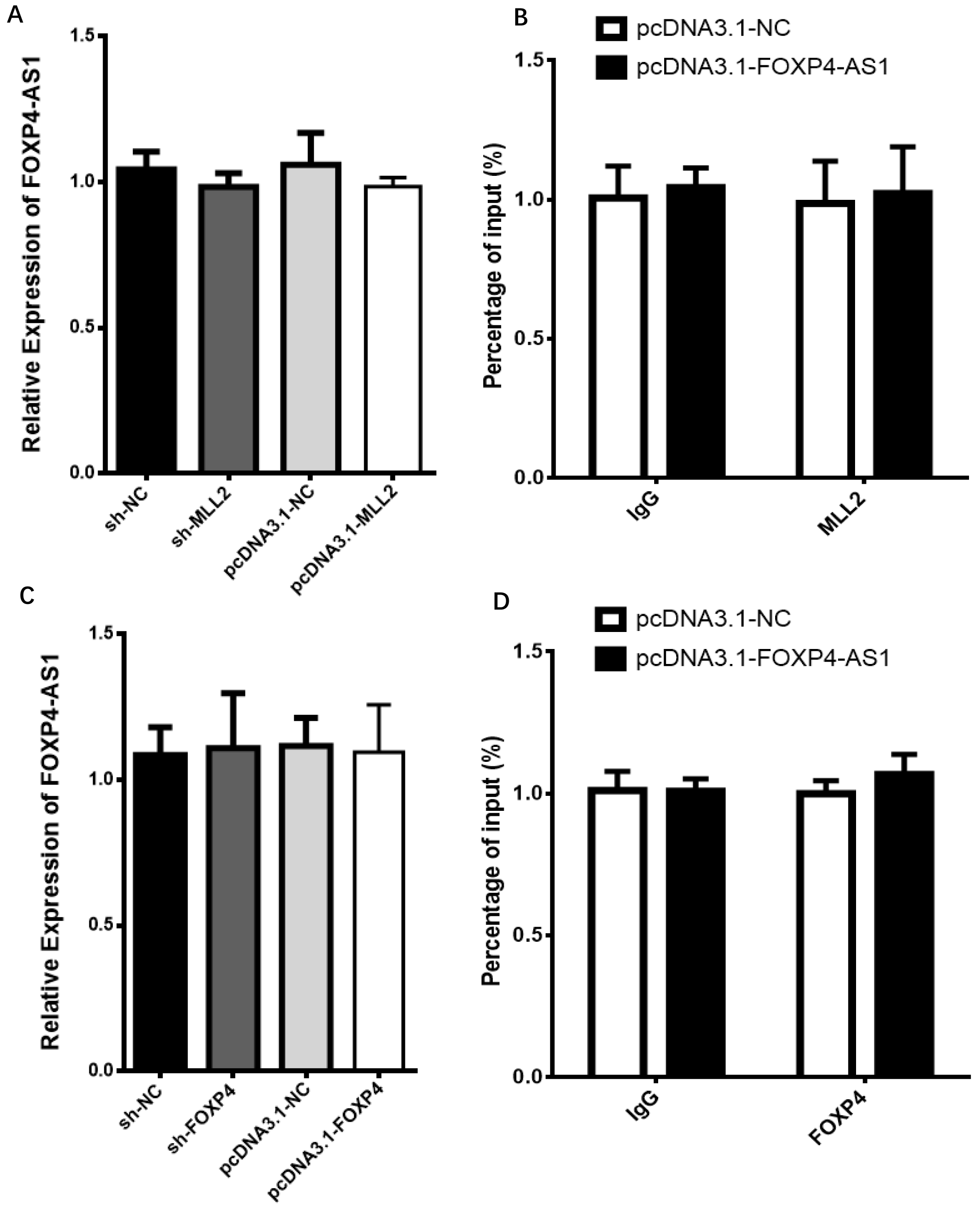
FIGURE S2 |** The influence of MLL2 and FOXP4 on FOXP4-AS1. **(A)** The expression of FOXP4-AS1with MLL2 by qRT-PCR. **(B)** The effect of MLL2 on FOXP4-AS1 promoter as determined by ChIP assay. **(C)** The expression of FOXP4-AS1with MLL2 by qRT-PCR. **(D)** The effect of MLL2 on FOXP4-AS1 promoter as determined by ChIP assay.
